# CBX6 is negatively regulated by EZH2 and plays a potential tumor suppressor role in breast cancer

**DOI:** 10.1038/s41598-018-36560-4

**Published:** 2019-01-17

**Authors:** Houliang Deng, Xiaowen Guan, Longcai Gong, Jianming Zeng, Hongjie Zhang, Mike Y. Chen, Gang Li

**Affiliations:** 1 Faculty of Health Sciences, University of Macau, Macau, China; 2 Division of Neurosurgery, Department of Surgery, City of Hope National Medical Center, Duarte, California, USA

## Abstract

Chromobox 6 (CBX6) is a subunit of Polycomb Repressive Complex 1 (PRC1) that mediates epigenetic gene repression and acts as an oncogene or tumor suppressor in a cancer type-dependent manner. The specific function of CBX6 in breast cancer is currently undefined. In this study, a comprehensive analysis of The Cancer Genome Atlas (TCGA) dataset led to the identification of CBX6 as a consistently downregulated gene in breast cancer. We provided evidence showing enhancer of zeste homolog 2 (EZH2) negatively regulated CBX6 expression in a Polycomb Repressive Complex 2 (PRC2)-dependent manner. Exogenous overexpression of CBX6 inhibited cell proliferation and colony formation, and induced cell cycle arrest along with suppression of migration and invasion of breast cancer cells *in vitro*. Microarray analyses revealed that CBX6 governs a complex gene expression program. Moreover, CBX6 induced significant downregulation of bone marrow stromal cell antigen-2 (BST2), a potential therapeutic target, via interactions with its promoter region. Our collective findings support a tumor suppressor role of CBX6 in breast cancer.

## Introduction

Polycomb group proteins (PcG) are important epigenetic regulators that function to maintain transcriptional repression. These proteins assemble into two major complexes in mammals, designated Polycomb Repressive Complex 1 (PRC1) and 2 (PRC2). The core components of the PRC2 complex include EZH1/2, SUZ12, EED, and RBAP46/48. Several other cofactors, such as JARID2, AEBP2 and Polycomb-like proteins (PCL1/PHF1, PCL2/MTF2, PCL3/PHF19) act as recruiters or modulators of PRC2 enzymatic activity^1–4^. PRC2 catalyzes the trimethylation of histone H3 lysine 27 (H3K27me3), a marker of transcriptional repression, via its methyltransferase subunits EZH1/2^5^. However, the components of PRC1 are considerably heterogeneous. For example, each of the *Drosophila* subunits has several homologs in mammals that assemble to form different PRC1 complex types. The core components of the canonical PRC1 complex include RING1A/B, PCGF, CBX, and PHC^6^. PRC1 monoubiquitinates histone H2A at lysine 119 (H2AK119ub) through the E3 ligase activity of RING1A/B, thereby contributing to gene silencing^7^. PRC2 and PRC1 are proposed to interact with each other to maintain gene repression. Canonically, PRC2 ‘writes’ H3K27me3 on chromatin of a given target gene locus, followed by binding of PRC1 to H3K27me3, leading to monoubiquitylation of H2A and subsequent chromatin compaction, and ultimately, gene repression^8^. Recent studies have shown that PRC1 can be recruited to target loci in a H3K27me3-independent manner and PRC1-dependent H2AK119ub1 recruits PRC2 to target genes^6,9^. PcG proteins are involved in multiple biological processes, including maintenance of cell identity, differentiation, proliferation, and cancer progression^10–15^.

*Drosophila* Polycomb protein (Pc) binds to H3K27me3 through a conserved N-terminal chromodomain^16^. Five orthologues of *Drosophila* Pc exist in mammals (CBX2, CBX4, CBX6, CBX7 and CBX8). Accumulating evidence supports critical roles of CBX proteins in tumorigenesis^17–19^. Remarkably, CBX proteins can act as either oncogenes or tumor suppressors in different cancer types. For example, CBX7 functions as a tumor suppressor and its expression is negatively associated with increased malignancy grades in bladder, pancreatic, glioma, breast, gastric, and colon carcinomas^20^. Conversely, CBX7 is overexpressed in prostate and ovarian cancer, implying an oncogenic role in these cancer types^20^. CBX8 acts as an oncogene in hepatocellular carcinoma (HCC) and promotes tumor growth and metastasis via activation of AKT/β-catenin signaling^21^, but suppresses cell migration, invasion and metastasis in esophageal squamous cell carcinoma (ESCC) and inhibits epithelial-mesenchymal transition (EMT) by repressing *SNAIL* expression^22^. The results of our primary study suggest that CBX6 is downregulated in glioblastomas and its overexpression reduces cell proliferative capacity^23^. However, frequent upregulation of CBX6 in HCC in association with promotion of cancer cell growth, both *in vitro* and *in vivo*, and poor prognosis has also been reported^24^. Therefore, the functions of individual CBX proteins in any cancer type should be investigated separately. While a number of CBX proteins, such as CBX2, 4, 7, and 8, have been shown to play vital roles in breast cancer progression^25–28^, the specific function of CBX6 in breast cancer progression remains to be elucidated.

Experiments from the current study demonstrated that *CBX6* expression was frequently downregulated in breast cancer. Notably, CBX6 was silenced epigenetically by EZH2 in a PRC2-dependent manner. In functional analyses, overexpression of CBX6 resulted in cell proliferation inhibition, induced cell cycle arrest and dramatically suppressed the migration and invasion capacities of MCF-7 cells. Furthermore, CBX6 induced significant downregulation of BST2 via binding to its promoter region to exert potential antitumor activity.

## Results

### CBX6 is frequently downregulated in human breast cancer

**Figure 1:**
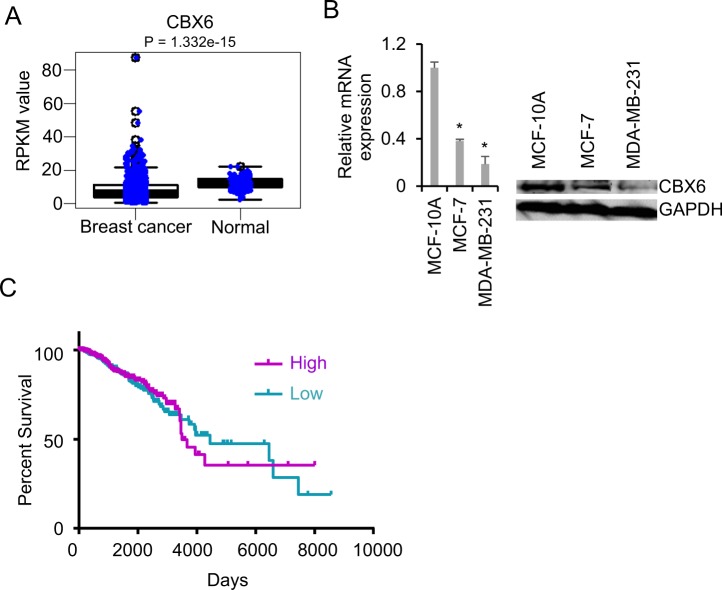
Chromobox 6 (*CBX6*) is downregulated in breast cancer. (**A**) mRNA expression of *CBX6* in breast cancer versus normal breast tissues. The data are retrieved from the RNA-Seq datasets of The Cancer Genome Atlas (TCGA). The expression values are presented as Reads Per Kilobase of transcript per Million mapped reads (RPKM). An unpaired two-tailed t-test was used to evaluate significant differences. P values are indicated at the top of the graph. (**B**) CBX6 expression in human non-tumorigenic breast epithelial (MCF-10A) and two human breast adenocarcinoma (MCF-7 and MDA-MB-231) cell lines. Left: qRT-PCR analysis of *CBX6* mRNA level in MCF-10A, MCF-7 and MDA-MB-231 cells. *GAPDH* was used for normalization of expression. Data are presented as mean ± S.D. from three independent experiments performed in triplicate. *P < 0.05. Right: Western blot analysis of CBX6 protein levels in MCF-10A, MCF-7 and MDA-MB-231 cells with GAPDH as a loading control. (**C**) Kaplan-Meier analysis of overall survival of breast cancer patients with different levels of CBX6 expression. Patient survival and gene expression data were downloaded from TCGA’s data portal and patients were split into two groups with the median CBX6 mRNA expression level as the cut-off.

To determine the specific role of CBX6 in breast cancer, we comprehensively analyzed The Cancer Genome Atlas (TCGA) dataset for aberrant expression of this gene (GSE62944). Significant downregulation of *CBX6* was observed in breast cancer tissues compared with controls, as shown in Fig. 1A. Gene expression profiling experiments have facilitated the identification of several subtypes of breast cancer, including luminal A, luminal B, HER2-enriched, and basal-like. Examination of the TCGA dataset revealed that *CBX6* is not differentially expressed in different subtypes of breast cancer (Supplementary Fig. S1A). *CBX6* expression was further analyzed in breast cancer samples with different histological grades. Our data showed similar expression profiles of *CBX6* at different stages (Supplementary Fig. S1B). To extend these observations, we tried to examine the expression of CBX6 by immunohistochemistry (IHC) in normal breast and breast cancer tissues. The signals detected using the CBX6 antibody (Millipore 09-030) are mainly located in the cytoplasm and connective tissues (Supplementary Fig. S2A). We interpreted that the IHC signal generated from this antibody was nonspecific, because CBX6 is primarily a nuclear protein as revealed by the immunofluorescence analysis of GFP-CBX6 fusion in MCF-7 cells (Supplementary Fig. S2B). The antibody recognized CBX6 immunoprecipitated from cell lysates (Supplementary Fig. S2C), and a band at the correct molecular weight of CBX6 in total cell lysates, but showed cross-reactivity with nonspecific bands of higher molecular weight. Next, the expression of CBX6 was assessed by qRT-PCR and by Western blotting using the antibody (Millipore 09-030) in a human non-tumorigenic epithelial cell line, MCF-10A, and two human breast adenocarcinoma cell lines, MCF-7 and MDA-MB-231. Consistently, CBX6 was significantly downregulated in breast cancer cells, compared with non-tumorigenic epithelial cells (Fig. 1B). In view of these findings, the association between CBX6 levels and clinical progression of breast cancer was further explored. Kaplan-Meier survival analysis of the TCGA breast cancer dataset showed the overall survival time did not significantly differ between patients with high CBX6 expression and low CBX6 expression when bifurcating gene expression at the median (HR = 0.94, P = 0.61) (Fig. 1C). However, survival analysis by the Kaplan-Meier plotter^29^, which curated the survival and gene expression data (Affymetrix microarray platform) of 5,143 breast cancer patients deposited in GEO^29^, showed patients with high CBX6 expression displayed significantly longer overall survival (OS) (HR = 0.78, P = 0.021) and recurrence-free survival (RFS) (HR = 0.73, P = 2.7e-08) than those with lower CBX6 expression^30^ (Supplementary Fig. S3). The discrepancy between the two datasets suggests the correlation between CBX6 expression and breast cancer patient survival needs further study.

### EZH2 negatively regulates CBX6 in breast cancer

**Figure 2:**
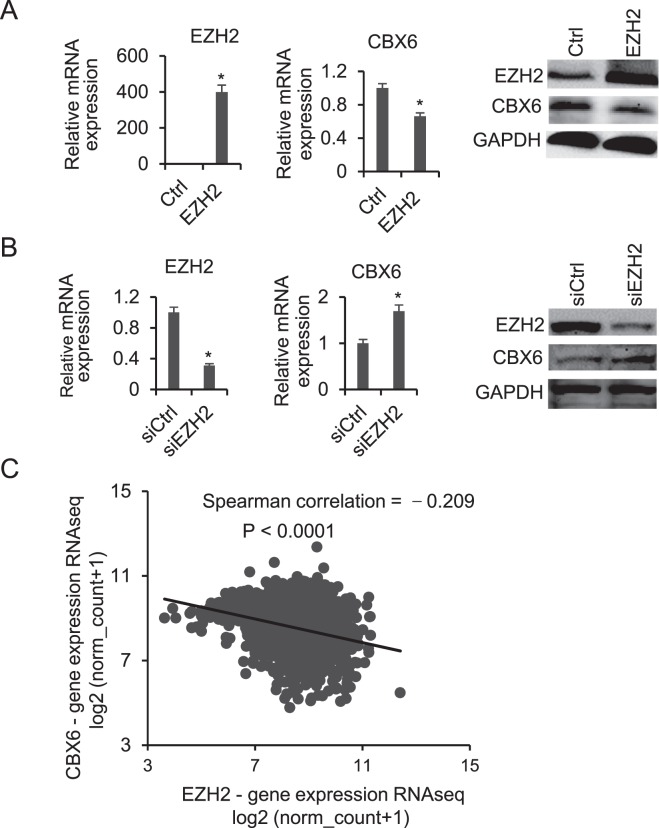
CBX6 is negatively regulated by EZH2. (**A**) Overexpression of EZH2 leads to downregulation of CBX6. Left and middle panels: qRT-PCR analysis of *EZH2* and *CBX6* mRNA in control (empty vector) and EZH2-overexpressing cells using *GAPDH* for normalization. Data are presented as means ± S.D. from three independent experiments performed in triplicate. *P < 0.05. Right panel: Western blot analysis of EZH2 and CBX6 protein levels in control (empty vector) and EZH2-overexpressing cells using GAPDH as a loading control. MCF-7 cells were transiently transfected with an empty or EZH2 vector for 48 hours, then cells were harvested, and total RNA and proteins were extracted for analysis. (**B**) Knockdown of EZH2 using siRNA leads to upregulation of CBX6. Left and middle panels: qRT-PCR analysis of *EZH2* and *CBX6* mRNA levels in siRNA control and EZH2 knockdown cells using *GAPDH* for normalization. Data are presented as means ± S.D. from three independent experiments performed in triplicate. *P < 0.05. Right: Western blot analysis of protein levels in siRNA control and EZH2 knockdown cells using GAPDH as a loading control. MCF-7 cells were transfected with control siRNA or siEZH2, total RNA and protein were extracted 48 h post-transfection for analysis. (**C**) Spearman’s rank correlation coefficient indicating a negative correlation between *CBX6* and *EZH2* mRNA levels in breast cancer tissues based on The Cancer Genome Atlas (TCGA) RNA-Seq data.

The mechanisms underlying CBX6 downregulation in breast cancer are not known at present. To identify the specific regulatory factors of CBX6 expression, we data-mined the ChIP-sequencing (ChIP-Seq) datasets deposited in the Gene Expression Omnibus (GEO). Interestingly, EZH2 and H3K27me3 peaks were detected at the CBX6 promoter in human mammary epithelial cells (HMEC) (Supplementary Fig. S4A), and a negative correlation between EZH2 and CBX6 expression was observed in MCF-10A, MCF-7, and MDA-MB-231 cells (Supplementary Fig. S4B). EZH2, which is highly expressed in breast cancer, promotes tumor progression and is associated with poorer patient outcome^31^. To further ascertain whether EZH2 is involved in regulation of CBX6, we performed overexpression and knockdown experiments in MCF-7 cells. CBX6 levels were markedly decreased upon EZH2 overexpression (Fig. 2A). Conversely, knockdown of EZH2 led to significant upregulation of CBX6 (Fig. 2B). Examination of the TCGA dataset further confirmed the negative correlation between EZH2 and CBX6 expression patterns in breast cancer (Spearman r = −0.209, P < 0.0001) (Fig. 2C).

### Regulation of CBX6 expression by EZH2 is PRC2-dependent

**Figure 3:**
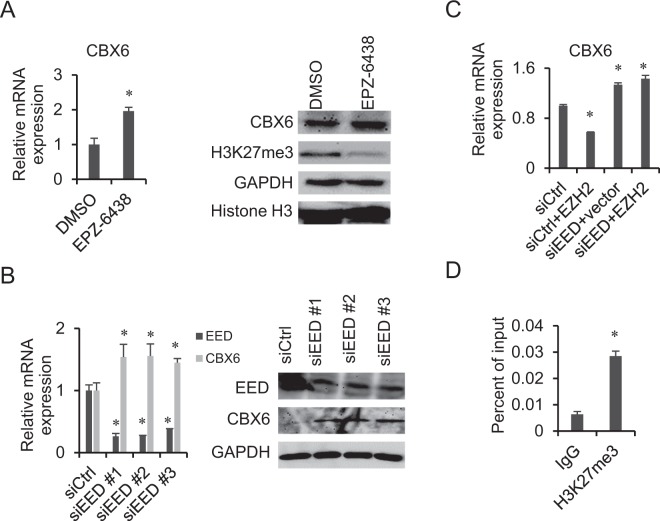
EZH2 negatively regulates CBX6 expression in a PRC2-dependent manner. (**A**) Treatment with the EZH2 inhibitor, EPZ-6438, induces downregulation of CBX6 in MCF-7 cells. Left: qRT-PCR analysis of *CBX6* mRNA in MCF-7 cells treated with DMSO or EPZ-6438. *GAPDH* was used for normalization. Data are presented as means ± S.D. from three independent experiments performed in triplicate. *P < 0.05. Right: Western blot analysis. MCF-7 cells were treated with 50 µM EPZ-6438 or DMSO for 5 days, then total RNA and protein were extracted for analysis. (**B**) Knockdown of EED is associated with upregulation of CBX6 in MCF-7 cells. Left: qRT-PCR analysis of *CBX6* mRNA in siRNA control (siCtrl) and *EED* knockdown cells using *GAPDH* for normalization. Data are presented as means ± S.D. from three independent experiments performed in triplicate. *P < 0.05. Right: Western blot analysis of siRNA control and EED knockdown cells with indicated antibodies. GAPDH was used as the loading control. MCF-7 cells were transfected with siEED or control siRNA at a final concentration of 50 nM using the Lipofectamine RNAiMAX transfection reagent. Total RNA and protein were extracted for analysis 48 h post-transfection. (**C**) EZH2 overexpression does not affect *CBX6* expression after knockdown of EED, as detected by qRT-PCR with *GAPDH* for normalization. Data are presented as means ± S.D. of three independent experiments performed in triplicate. *P < 0.05. MCF-7 cells were transfected with control siRNA or siEED for 24 h, followed by transient transfection of EZH2 or empty vector for 24 h. Total RNA was then extracted for analysis. (**D**) Enrichment of H3K27me3 at *CBX6* promoter as detected by ChIP-qPCR, *P < 0.05.

The gene regulatory activity of EZH2 can either be PCR2-dependent or -independent. Accordingly, we investigated whether or not EZH2-mediated downregulation of CBX6 is influenced by PRC2. To this end, MCF-7 cells were treated with the EZH2 inhibitor, EPZ-6438. As shown in Fig. 3A, following EPZ-6438 treatment, the H3K27me3 level was dramatically decreased and that of CBX6 was significantly increased in MCF-7 cells. EED, a core component of PRC2, is indispensable for PRC2 activity. We employed three different siRNAs for EED knockdown in MCF-7 cells, which all led to a marked increase in CBX6 expression (Fig. 3B). In addition, EZH2 overexpression no longer affected CBX6 expression after EED knockdown in MCF-7 cells (Fig. 3C). Furthermore, H3K27me3 enrichment at the CBX6 promoter was detected using ChIP-qPCR (Fig. 3D), indicating regulation of CBX6 expression by EZH2 is PRC2-dependent.

### CBX6 inhibits proliferation and induces cell cycle arrest in MCF-7 cells

**Figure 4:**
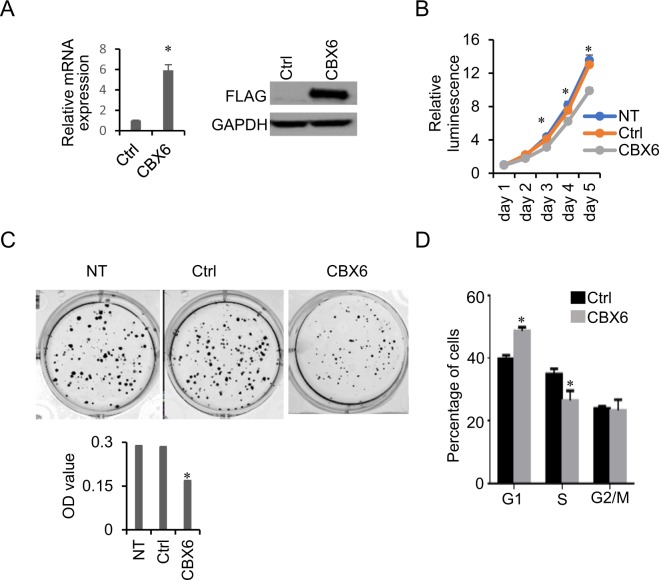
CBX6 inhibits cell proliferation and induces cell cycle arrest in MCF-7 cells. (**A**) Stable overexpression of CBX6-FLAG in MCF-7 cells. MCF-7 cells were transfected with CBX6-FLAG vector, then subjected to 1 mg/mL G418 selection to obtain MCF-7 cells stably overexpressing CBX6. qRT-PCR and western blot analysis were conducted to confirm the overexpression of CBX6-FLAG in the stable cell line. MCF-7 cells were transfected with an empty vector to serve as a control (Ctrl). (**B**) Non-transfected (NT) MCF-7 cells, MCF-7 cells stably transfected with the empty vector (Ctrl), or CBX6-FLAG (CBX6) were seeded into 96-well plates. Cell proliferation was assessed using the CellTiter-Glo® Luminescent Cell Viability Assay (Promega) for 5 days. A representative of three independent experiments performed in triplicate is shown. *P < 0.05. (**C**) Colony formation assay. Non-transfected (NT) MCF-7 cells, MCF-7 cells stably transfected with the empty vector (Ctrl), or CBX6-FLAG (CBX6) were seeded into 6-well plates for 15 days. Colonies were stained with 0.1% crystal violet and methanol was added to solubilize the dye. Optical density (OD) values of absorbance at 540 nm was measured. Data are representative of three independent experiments performed in triplicate. *P < 0.05. (**D**) CBX6 induces cell cycle arrest. Control (empty vector) and CBX6-overexpressing MCF-7 cells were seeded into 6-well plates and synchronized via starvation (without serum) for 24 h. Cells were then cultured under normal conditions for 24 h and analyzed by flow cytometry. Data are representative of three independent experiments performed in triplicate, *P < 0.05.

To determine the biological significance of CBX6 dysregulation, we generated stable MCF-7 cell lines expressing vector alone or FLAG-tagged CBX6 (Fig. 4A) and examined the effects of CBX6 overexpression on cell growth. As shown in Fig. 4B, CBX6-overexpressing MCF-7 cells grew moderately slower, compared with control cells transfected with the empty vector. Moreover, CBX6-overexpressing cells formed fewer and smaller colonies relative to the control group (empty vector) (Fig. 4C upper panel). This effect was further confirmed by measuring absorbance after solubilization of cell-bound crystal violet (Fig. 4C, lower panel). To assess whether the lower growth rate of CBX6-overexpressing cells is attributable to alterations in the cell cycle, we examined cell cycle distribution via flow cytometry. Our data showed a higher percentage of CBX6-overexpressing cells in the G1 phase and a lower percentage in the S phase, compared with control cells expressing empty vector (Fig. 4D).

### CBX6 inhibits migration and invasion of MCF-7 cells

**Figure 5:**
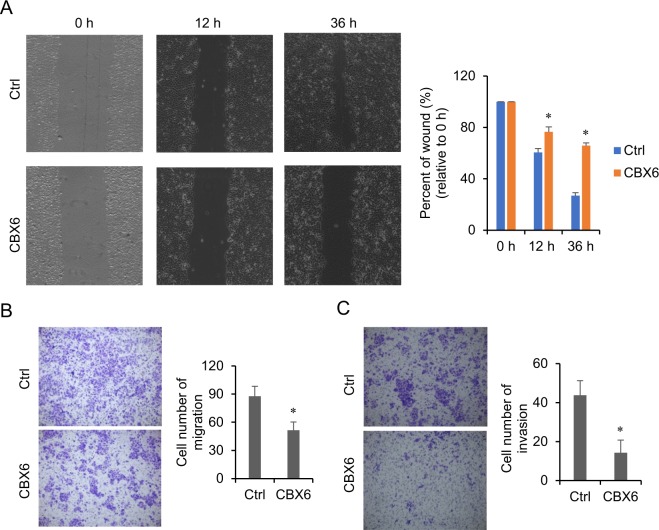
CBX6 inhibits migration and invasion of MCF-7 cells. (**A**) Wound healing assay. The wound gaps were generated after cells reached 90% confluence. Images of cells were obtained at 0, 12, and 36 h post-wounding with the EVOS FL cell imaging system (Invitrogen). Representative images of the wound healing assay were shown on the left. The wound area was measured, and percent of initial wound area was compared between MCF-7 cells stably transfected with the empty vector (Ctrl), or CBX6-FLAG (CBX6) (right panel). *P < 0.05. (**B**,**C**) *Transwell* invasion and migration assay. Representative images of migrated (**B**) or invaded (**C**) cells were shown on the left. Cell numbers were quantified by counting ten random fields at ×200 magnification. MCF-7 cells stably transfected with CBX6-FLAG (CBX6) have decreased abilities of migration and invasion, compared to the cells stably transfected with the empty vector (Ctrl). *P < 0.05.

Metastasis is an essential hallmark of cancer. To investigate the effects of CBX6 on metastasis and invasion, we performed cell migration and invasion assays *in vitro*. In the wound healing assay, CBX6-overexpressing MCF-7 cells showed delayed wound healing closure, compared to control cells (Fig. 5A). Similarly, CBX6 overexpression induced a dramatic reduction in migration and invasion of MCF-7 cells in a *Transwell* assay (Fig. 5B,C).

### Gene expression changes induced by CBX6 overexpression

**Figure 6:**
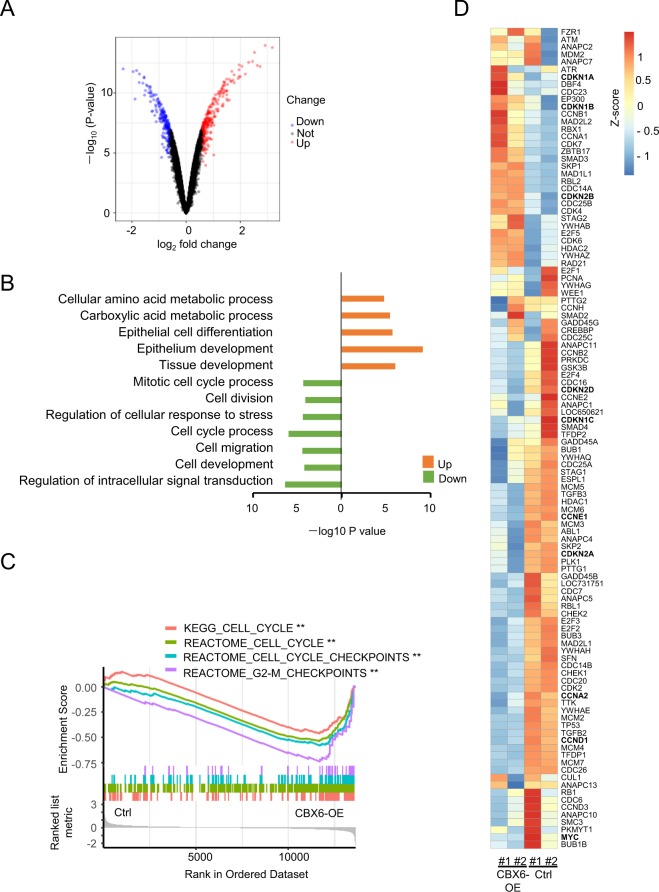
Effects of CBX6 overexpression on gene expression in MCF-7 cells. (**A**) Gene expression was examined by HumanHT-12 v4Expression BeadChip microarrays (Illumina). The volcano plot shows statistical significance (−log_10_ (P-value) plotted against log_2_ fold change of genes for CBX6-overexpressing cells against control (empty vector) cells. Differentially expressed genes were selected based on criteria of P < 0.05 and absolute log_2_ fold change >0.6. (**B**) Gene ontology (GO) analysis of significantly altered genes in CBX6-overexpressing cells. (**C**) Gene set enrichment analysis (GSEA) of gene expression profiles of MCF-7 cells overexpressing CBX6 (CBX6-OE) versus control. Shown are results using REACTOME “Cell Cycle”, “Cell Cycle Checkpoints”, “G2-M Checkpoints” and Kyoto Encyclopedia of Genes and Genomes (KEGG) “Cell Cycle” as the interrogating gene sets. **P ≤ 0.01. (**D**) The expression heatmap of genes included in the gene set of KEGG “Cell Cycle” in MCF-7 cells overexpressing CBX6 (CBX6-OE), and control MCF-7 cells (Ctrl) which are stably transfected with the empty vector.

To clarify the mechanisms underlying CBX6 activity in breast cancer progression, MCF-7 cells stably overexpressing the gene were subjected to microarray analysis. In total, 525 genes were differentially expressed between CBX6-overexpressing and vector control cells, among which 234 were downregulated (log_2_ fold change <−0.6, P < 0.05) and 291 were upregulated (log_2_ fold change >0.6, P* < *0.05) in CBX6-overexpressing cells (Fig. 6A; Supplementary Table S2). Gene ontology analysis revealed that upregulated genes in CBX6-overexpressing cells are involved in epithelial cell differentiation and epithelium development whereas downregulated genes participate in the cell cycle, cell division, cell migration, cell development, regulation of intracellular signal transduction and cellular response to stress (Fig. 6B).

To gain more insights into the functional consequences of restoring CBX6 expression in MCF7 cells, we performed gene set enrichment analysis (GSEA), and found that multiple cell cycle genes, included in the gene matrices of the Kyoto Encyclopedia of Genes and Genomes (KEGG) Cell cycle, REACTOME cell cycle, REACTOME cell cycle checkpoints and REACTOME G2_M checkpoints, are downregulated in CBX6-overexpressing (CBX6-OE) MCF-7 cells (Fig. 6C). Specifically, several cyclins including G1/S Cyclin *CCND1* and *CCNE1* are downregulated in CBX6-OE MCF-7 cells, consistent with the slight G1-arrest in CBX6-OE cells. Meanwhile, the cyclin-dependent kinase inhibitor gene, including *CDKN1B* (p27), *CDKN1A* (p21) are upregulated in CBX6-OE MCF-7 cells. *CDKN2A* (p16^INK4A^/p14^ARF^) is a known Polycomb target gene^32^; its expression is downregulated in CBX6-OE MCF-7 cells. Paradoxically, *CDK2B*, the neighboring gene of *CDKN2A*, is upregulated in CBX6-OE MCF-7 cells. *MYC*, the oncogene which amplifies the output of the existing gene expression program^33^, and promotes tumorigenesis by regulating cell growth, proliferation and metabolism^34^, is downregulated by CBX6 overexpression (Fig. 6D). All these results indicate that CBX6 governs a complex gene expression program.

### BST2 is directly downregulated by CBX6

PcG proteins have context-dependent actions on gene expression^35^. We reasoned high-confidence direct targets of CBX6 should have a greater tendency to be modulated by CBX6 in different cell line models. To ascertain potentially significant genes in the CBX6-associated tumorigenesis, we performed microarray analysis on a U251MG glioma cell line overexpressing CBX6 that we previously established (Deng *et al*., unpublished results). Searching for genes affected led to the identification of multiple genes repressed by CBX6’s overexpression in both cell lines, including bone marrow stromal cell antigen 2 (BST2). *BST2*, which encodes a type II integral membrane protein that inhibits the release of enveloped viruses through its homodimerization^36,37^, is overexpressed in multiple cancers including breast cancer^37^. BST2 was demonstrated to promote tumor survival, invasion and metastasis through a plethora of mechanisms^37,38^.

**Figure 7:**
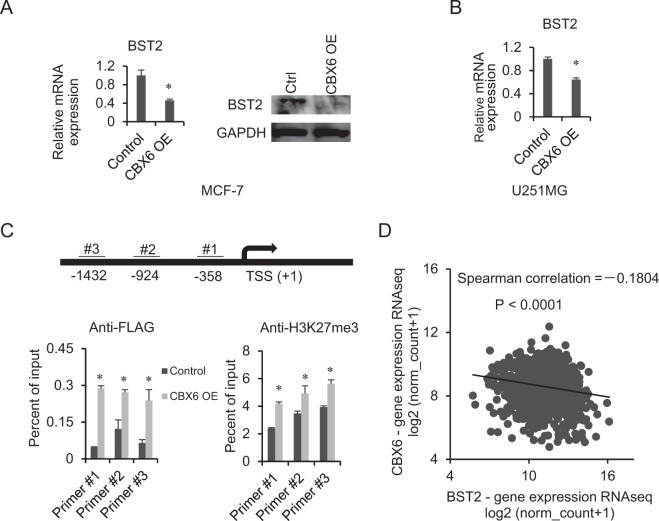
CBX6 directly downregulates the expression of Bone Marrow Stromal cell antigen 2 (BST2). (**A**) CBX6 downregulates BST2 expression in MCF-7 cells. Left: qRT-PCR analysis of *BST2* mRNA in the control (empty vector) and CBX6-overexpressing (CBX6-OE) cells using *GAPDH* for normalization. Data are presented as means ± S.D. from three independent experiments performed in triplicate. *P < 0.05. Right: Western blot analysis of BST2 in control (empty vector) and CBX6-overexpressing cells (CBX6-OE) using GAPDH as a loading control. (**B**) CBX6 downregulates BST2 expression in U251MG cells. qRT-PCR analysis of *BST2* mRNA levels in control (empty vector) and CBX6-overexpressing cells using *GAPDH* for normalization. Data are presented as means ± S.D. from three independent experiments performed in triplicate. *P < 0.05. (**C**) Enrichment of CBX6 and H3K27me3 at the *BST2* promoter detected via ChIP-qPCR. Top: The locations of primers at the *BST2* promoter. Bottom: Enrichment of CBX6 and H3K27me3 at the *BST2* promoter in control (empty vector) and CBX6-overexpressing cells. Data are presented as a percentage of the input, and as means ± S.D. from three independent experiments performed in triplicate. *P < 0.05. (**D**) Spearman’s rank correlation coefficient indicating a negative correlation between CBX6 and BST2 mRNA levels in breast cancer tissues based on The Cancer Genome Atlas (TCGA) RNA-Seq data.

We applied qRT-PCR and western blot analyses to confirm the regulation of BST2 by CBX6. As expected, overexpression of CBX6 in MCF-7 cells was associated with decreased levels of BST2 mRNA and protein (Fig. 7A). Examination of the CBX6-OE U251MG cells and its control revealed that overexpression of CBX6 also caused a significant decrease in BST2 in U251MG cells (Fig. 7B). We further determined whether CBX6 directly targets BST2. As shown in Fig. 7C, both CBX6 and H3K27me3 were significantly enriched at the BST2 promoter in CBX6-OE MCF-7 cells. Moreover, examination of TCGA dataset disclosed a marked increase in BST2 expression in breast cancer (Supplementary Fig. S5) and CBX6 and BST2 were negatively correlated in breast cancer (Spearman r = −0.1804, P < 0.0001) (Fig. 7D). Based on these findings, we propose that CBX6 potentially plays a tumor suppressor role through repression of BST2 in breast cancer.

## Discussion

### PcG-mediated gene silencing plays a critical role in cancer development

CBX6, a subunit of PRC1, reads the crucial epigenetic marker, H3K27me3, written by PRC2. Recent studies have reported that CBX6 assembles into PRC1 and its knockdown via shRNA induces spontaneous differentiation of embryonic stem cells (ESC), supporting a role as an essential regulator of ESC identity^39^. Ectopic expression of CBX6 is reported to block reprogramming^40^ and its dysregulation implicated in different types of malignancies^23,24^. Experiments in the current study revealed significant downregulation of CBX6 in breast cancer. CBX6 inhibited cell proliferation and induced cell cycle arrest in MCF-7 *in vitro*. Moreover, cell migration and invasion rates were significantly reduced upon CXB6 overexpression.

We examined changes in gene expression profiles caused by CBX6 overexpression via microarray analysis. Consistent with functional experimental results, genes downregulated in association with CBX6 overexpression are involved in the cell cycle, division and migration. For example, CBX6 represses expressions of G1/S Cyclin CCND1 and CCNE1 in MCF-7 cells. Both CCND1 and CCNE1 were reported to be repressed by PcG proteins: CBX7, a paralog of CBX6, binds the CCNE1 prompter and represses the expression of CCNE1 partly through HDAC2 in mice and human lung carcinomas and gliomas^41,42^; JARID2, a co-factor facilitating genomic targeting of PRC2, represses CCND1’s expression in cardiomyocytes and leukemia^43–45^. In this study, we also found CBX6 repressed MYC expression in MCF-7 cells. Kaur *et al*. reported that MYC limits its own expression by a feedback loop mediated by EZH2^46,47^, the finding here suggests CBX6 might participate in the process. In addition to amplifying transcription, paradoxically, MYC represses discrete sets of genes through direct and indirect mechanisms^48^. Best characterized MYC-repressed genes include CDKN1A, CDKN1B and CDKN2B^48^, all of which are upregulated in the MCF-7 cell upon CBX6 over-expression, suggesting a potential CBX6-MYC-CDK inhibitors pathway regulating cell cycle progression. Nevertheless, the results here indicate that CBX6 regulates complex regulatory circuits, further research is needed to ascertain the effects of CB6 on gene expression, elucidate the detailed mechanisms by which CBX6 controls gene expression, directly or indirectly.

In addition to breast cancer, CBX6 is reported to be downregulated in glioblastomas although the underlying mechanisms are still unknown. EZH2 is highly expressed in multiple cancers, including glioblastoma and breast cancer. Moreover, high expression of EZH2 is associated with aggressive disease and poor outcome^49–51^. To date, several small molecule inhibitors targeting EZH2 have been investigated in preclinical and clinical trials^52^. PcG proteins are suggested to auto-regulate their own expression in human embryonic fibroblasts^53^. Overexpression and knockdown experiments in the current study showed that EZH2, the catalytic subunit of PRC2, repressed the expression of CBX6. Earlier studies suggest that gene expression regulated by EZH2 is either PRC2-dependent or independent^54,55^. Here, we observed upregulation of CBX6 expression by the EZH2 inhibitor EPZ-6438. Knockdown of EED led to upregulation of CBX6 in MCF-7 cells. Additionally, EZH2 overexpression did not affect CBX6 expression after knockdown of EED in MCF-7 cells. Our data collectively indicate that downregulation of CBX6 by EZH2 requires intact PRC2 activity.

Bone marrow stromal cell antigen 2 (BST2), also known as CD317 or tetherin, is a lipid raft-associated type 2 transmembrane glycoprotein. BST2 promotes activation of NF-kB, leading to the production of proinflammatory factors involved in inhibition of viral replication^56,57^ and additionally implicated in blocking virion release via physically tethering to the cell surface^58^. BST2 may be involved in cell-to-cell interactions in view of the finding that purified extracellular domains of the protein inhibit the adhesion of human monocytes to HUVECs^59^. Inflammatory responses and cell adhesion contribute to tumorigenesis, indicating a role in cancer progression. Accumulating studies have provided evidence that BST2 is involved in tumor progression in many cancer types, including breast cancer^60–67^. Yi *et al*. demonstrated that BST2 is upregulated in tamoxifen-resistant breast cancer cells and enhances the invasion and migration capacities of tumor cells. Mahauad-Fernandez’s research group reported that knockdown of BST2 inhibits mammary tumor growth and metastasis, both *in vitro* and *in vivo*, and high BST2 expression in breast tumors is positively associated with tumor size and aggressiveness as well as poor patient survival^63^. In addition, monoclonal antibodies against BST2 possess significant antitumor activity in lymphoma and endometrial cancers^68,69^. In view of the combined results, BST2 is proposed as an ideal therapeutic target for breast cancer. In the current study, CBX6 significantly suppressed BST2 expression in both MCF-7 and U251MG cells, implying that this process is independent of cell type. Additionally, CBX6 bound directly to the BST2 promoter and increased enrichment of H3K27me3.

In conclusion, our experiments provide evidence of significant downregulation of CBX6 expression in breast cancer. CBX6 was negatively regulated by EZH2 in a PRC2-dependent manner. Overexpression of CBX6 inhibited the proliferation and metastasis capacity in breast cancer cells *in vitro*, altered the expression of genes involved in cell cycle regulation and other pathways. Moreover, CBX6 exerted a marked suppressive effect on expression of BST2 through binding to its promoter and altering histone modification. To our knowledge, this is the first study to demonstrate a tumor suppressor role of CBX6 in breast cancer.

## Methods

### Bioinformatic analysis

mRNA expression profiles and clinical information on the 1119 breast carcinomas and 113 normal samples were obtained from The Cancer Genome Atlas (TCGA) data portal. Gene set enrichment analysis^70^ was performed as described^71^. Kaplan-Meier survival analyses were performed using GraphPad Prism 6 or the Kaplan-Meier plotter (www.kmplot.com)^29^.

### Cell culture and drug treatment

MCF-7 and MDA-MB-231 were kindly provided by Professor Edwin Chong Wing Cheung (Faculty of Health Sciences, University of Macau) and MCF-10A by Professor Lijun Di (Faculty of Health Sciences, University of Macau). C2C12 was purchased from the Cell Resources Center of Shanghai Institute for Biological Sciences, Chinese Academy of Sciences (Shanghai, China). MCF-7, MDA-MB-231 and C2C12 cells were cultured in DMEM (Gibco) containing 10% FBS. MCF10A cells were cultured in DMEM supplemented with 10% FBS, 0.5 µg/mL hydrocortisone (Sigma), 10 µg/mL insulin (Thermo Fisher), and 20 ng/mL hEGF (Thermo Fisher). All cells were tested negative for mycoplasma contamination and were maintained at 37 °Cand 5% CO_2_. For drug treatment, MCF-7 cells were seeded in 6-well plates and incubated overnight, followed by treatment with 50 µm EPZ6438 (Selleck Chemicals). Control cells were treated with 0.1% DMSO. All cells were treated for 5 consecutive days followed by extraction of protein and RNA for analysis.

### Plasmids and transfection

Human CBX6-Myc-DDK cloned into pCMV6_Entry was purchased from OriGene (RC204166). Human EZH2 was amplified using cDNA derived from MCF-7 cells as a template and cloned into pcDNA3.2/GW/D-TOPO expression vector (Invitrogen). Expression vectors were transfected into MCF-7 cells with Lipofectamine 2000 reagent (Invitrogen) according to the manufacturer’s instructions. Cells transfected with the corresponding empty expression vector served as the controls. At 48 h post-transfection, protein and RNA were isolated for analysis. For generating a CBX6-expressing stable cell line, 1 mg/mL G418 (Gibco) was added to the culture medium to screen for stable clones. Expression of CBX6 mRNA and protein was verified via qRT-PCR and western blot analysis, respectively. For knockdown experiments, a pool of siRNAs targeting EZH2 (Santa Cruz Biotechnology; sc35312) and three EED siRNAs (Integrated DNA Technologies (IDT)) were employed. MCF-7 cells were transfected with test or control siRNA at a final concentration of 50 nM using Lipofectamine RNAiMAX transfection reagent (Invitrogen) according to the manufacturer’s instructions. At 48 h post-transfection, protein and RNA were harvested for analysis.

### Cell proliferation assay

MCF-7 cells (2 × 10^3^) were plated in 96-well plates in 100 µL medium. Cell proliferation was analyzed using the CellTiter-Glo® Luminescent Cell Viability Assay (Promega) for five consecutive days.

### Colony formation assay

MCF-7 cells were seeded into 6-well plates at a density of 500 cells per well. At 15 days after seeding, each well was stained with 0.1% crystal violet and methanol added to solubilize the dye. Absorbance at 540 nm was read using a Spark Multimode microplate reader (Tecan).

### Flow cytometry analysis of the cell cycle

MCF-7 cells were seeded into 6-well plates and synchronized via starvation (without serum) for 24 h, followed by culturing under normal conditions for 24 h. Cells were harvested and stained using cell cycle and apoptosis analysis kits (Beyotime) and analyzed with a FACS scan flow cytometer (BD Biosciences). The relative ratios of G1, S, and G2 phases were analyzed using the FlowJo 2.8 software.

### Wound healing assay

MCF-7 cells were seeded into 6 well-plates. After cells reached 90% confluence, cross lines were generated using a sterile pipette tip. Images of cells were obtained after 0, 12 and 36 h post-wounding using the EVOS FL cell imaging system (Thermo Fisher).

### *Transwell* assay

The *Transwell* assay was performed in 24-well chambers (Corning). For the migration assay, 2.5 × 10^5^ cells were resuspended in serum-free medium and added to the upper chamber. The bottom chamber was filled with 500 μL culture medium containing 10% FBS. After culturing for 24 hours, cells on the lower surface of the membrane were stained with 0.1% crystal violet and photographed using the Carl Zeiss Axio Observer microscope. For the invasion assay, the upper chamber surface of the basement membrane of the *Transwell* was coated with Matrigel (Corning). Aliquots of cells (1 × 10^6^) were resuspended in serum-free medium and added to the upper chamber. The bottom chamber was filled with 500 μL culture medium containing 10% FBS. After culturing for 36 hours, cells on the lower surface of the membrane were stained with 0.1% crystal violet and photographed using the Carl Zeiss Axio Observer microscope.

### Total RNA extraction

Total RNA was extracted using the TRIzol reagent (Invitrogen). Genomic DNA was digested with an RNase-free DNase kit (Qiagen) and subsequently purified using the RNeasy Mini Kit (Qiagen) following the manufacturer’s instructions. The concentration and quality of RNA were determined using an Agilent 2100 Bioanalyzer (Agilent Technologies).

### Gene expression profiling and analysis

Total RNA was amplified and labeled using the TargetAmp™-Nano Labeling Kit for Illumina Expression BeadChip (Epicentre Biotechnologies). Labeled cRNA was purified with the RNeasy mini kit (Qiagen) and hybridized on HumanHT-12 v4 Expression BeadChip microarrays (Illumina) according to the manufacturer’s protocol. Hybridized arrays were scanned using the Illumina iScan, and the image data was extracted using the Illumina Genome Studio software. Gene ontology analysis was conducted using the PANTHER Overrepresentation Test (http://www.geneontology.org/).

### Quantitative real-time reverse transcription–polymerase chain reaction (qRT-PCR)

Total RNA (1 μg) was used to synthesize cDNA using a PrimeScript™ RT Reagent Kit with gDNA Eraser (Takara). Quantitative PCR (qPCR) was conducted using the iTaq™ Universal SYBR® Green Supermix (Bio-Rad) in the CFX 96 thermocycler (Bio-Rad). Assays were performed in triplicate and repeated three times. The relative expression level of the gene of interest was normalized to that of GAPDH and calculated according to the 2−ddCt method. The primers used in this study are presented in Supplementary Table S1.

### Western blot

Whole cell lysates were harvested using RIPA Lysis and Extraction Buffer (Invitrogen) supplemented with the protease inhibitor cocktail (Sigma). The extracted protein was separated via SDS-PAGE and transferred to nitrocellulose membranes (Pall Corporation). Membranes were blocked with 5% non-fat milk and incubated with the following antibodies: CBX6 (Millipore 09-030), FLAG (Sigma-Aldrich F1804), EZH2 (Cell Signaling Technology 5246 S), H3K27me3 (Abcam ab6002), EED (Millipore 05-1320), BST2 (Santa Cruz Biotechnology sc-390719), GAPDH (Cell Signaling Technology 5174 s), H3 (Abcam ab1791), followed by horseradish peroxidase-conjugated secondary antibody (Jackson ImmunoResearch, 1:5000) for 60 min at room temperature. Signals were detected with the enhanced chemiluminescence (ECL) detection system (Thermo Fisher Scientific).

### Chromatin immunoprecipitation (ChIP)

Chromatin immunoprecipitation (ChIP) was performed using the EZ-Magna ChIP™ A/G Chromatin Immunoprecipitation Kit (Millipore) in accordance with the manufacturer’s instructions. Briefly, cells were cross-linked with 1% paraformaldehyde and sonicated to obtain fragments between 0.2 and 0.5 kb. Chromatin was incubated with anti-FLAG (Sigma-Aldrich F1804), anti-H3K27me3 (Millipore 07-449) or control mouse IgG (Cell Signal Technology 5415) antibodies and protein A/G beads overnight. DNA was extracted and used for ChIP-qPCR analysis. Enrichment levels are presented as a percentage of input chromatin. The primers are presented in Supplementary Table S1.

## Electronic supplementary material

Supplementary Information

Supplementary Table S2
