# Supplementary material for: CBX6 is negatively regulated by EZH2 and plays a potential tumor suppressor role in breast cancer
Source: Sci Rep. 2019 Jan 17;9:197. doi: 10.1038/s41598-018-36560-4 (PMC6336801; doi:10.1038/s41598-018-36560-4)

Supplementary Information for:

CBX6 is negatively regulated by EZH2 and plays a potential tumor suppressor role in breast cancer

Houliang Deng[1], Xiaowen Guan[1], Longcai Gong[1], Jianming Zeng[1], Hongjie Zhang[1], Mike Y. Chen[2], Gang Li[1*]

[1]*Faculty of Health Sciences, University of Macau, Macau, China.*

[2]*Division of Neurosurgery, Department of Surgery, City of Hope National Medical Center, Duarte, California, USA.*

*Corresponding Author:

Gang Li, Ph.D.
Assistant Professor
Faculty of Health Sciences, University of Macau
E12-3014, Avenida da Universidade, Taipa, Macau, China
Tel: +853-8822-4212
Fax: +853-8822-2314
E-mail: gangli@umac.mo

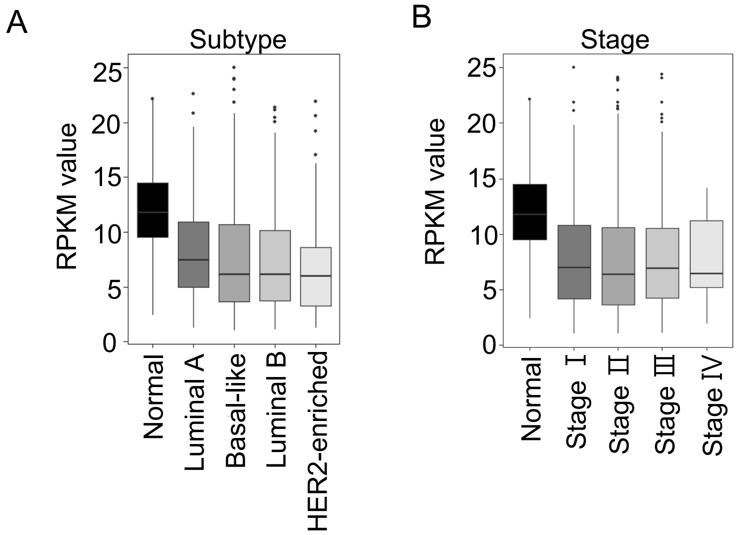

Figure S1

CBX6 mRNA levels in different subtypes and stages of breast cancer. (**A**) CBX6 mRNA levels in different subtypes of breast cancer. (**B**) CBX6 mRNA levels in different stages of breast cancer. RPKM: Reads Per Kilobase of transcript per Million mapped reads.

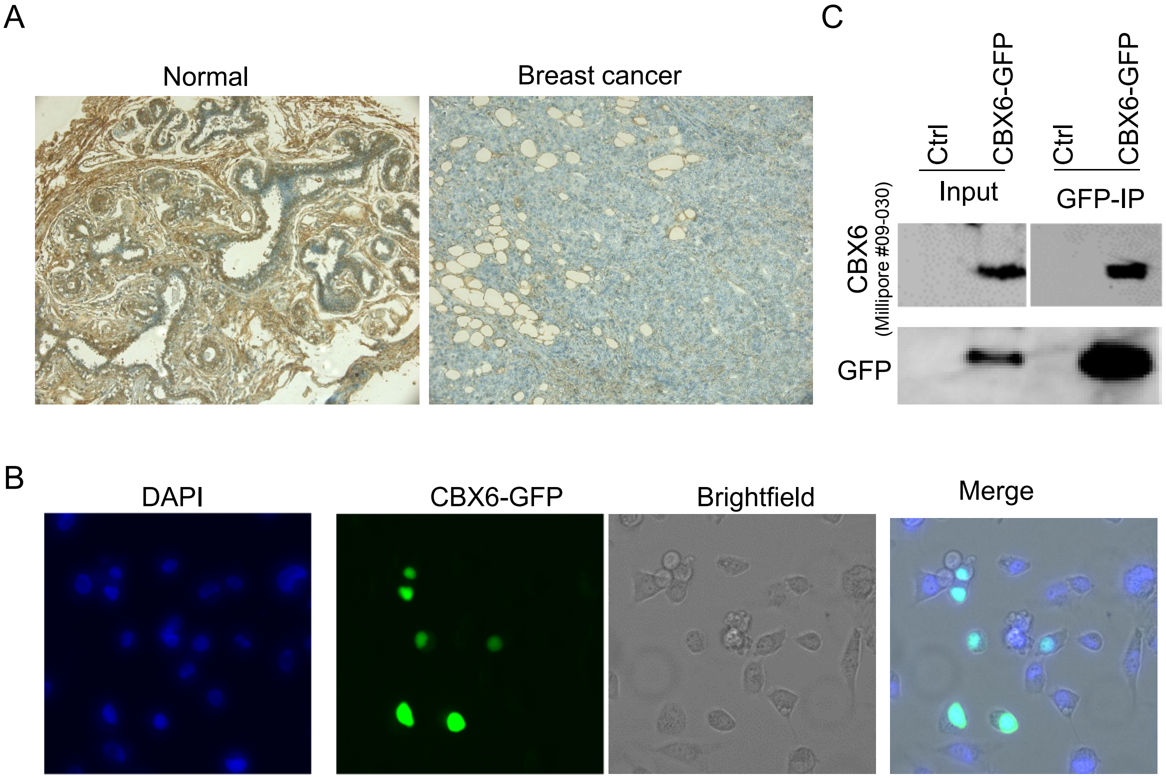

Figure S2

Validating the antibody against CBX6 from Millipore (#09-030). (**A**) Immunostaining of CBX6 in normal breast and breast cancer tissues. Tissue sections were incubated with the antibody against CBX6 (Millipore (#09-030), stained using the SuperPicture 3rd Gen IHC Detection Kit (Thermo Fisher Scientific) according to the manufacturer's protocol, then counterstained with hematoxylin. Cytoplasmic staining signals or putative non-specific signals were observed. (**B**) CBX6 is localized in the nucleus. CBX6-GFP was transiently transfected into MCF-7 cells, cell nuclei were stained with the Hoechst 33342 dye. Images were captured with ZOE™ Fluorescent Cell Imager (Bio-Rad). (**C**) The antibody (Millipore 09-030) recognizes CBX6. A construct encoding a CBX6-GFP fusion protein was transient-transfected into 293T cells for 48 hours, and CBX6-GFP was immunoprecipitated using GFP-Trap® resin (ChromoTek #gta-100) and detected by Western blotting using the antibody indicated.

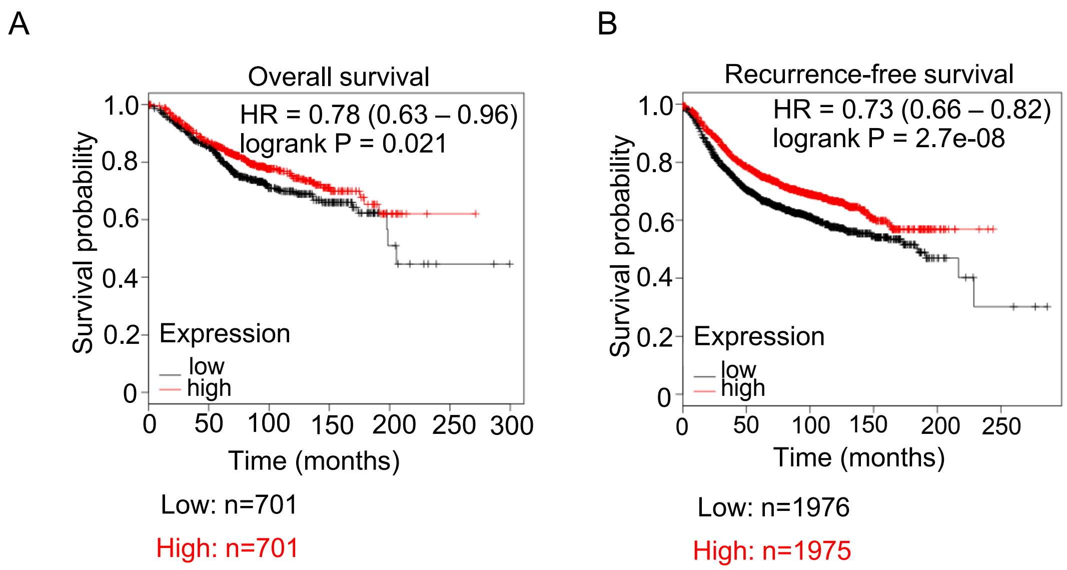

Figure S3

High mRNA expression of CBX6 is significantly associated with longer overall survival (OS) (**A**) and recurrence-free survival (RFS) (**B**) in breast cancer patients analyzed using the Kaplan-Meier plotter (www.kmplot.com).

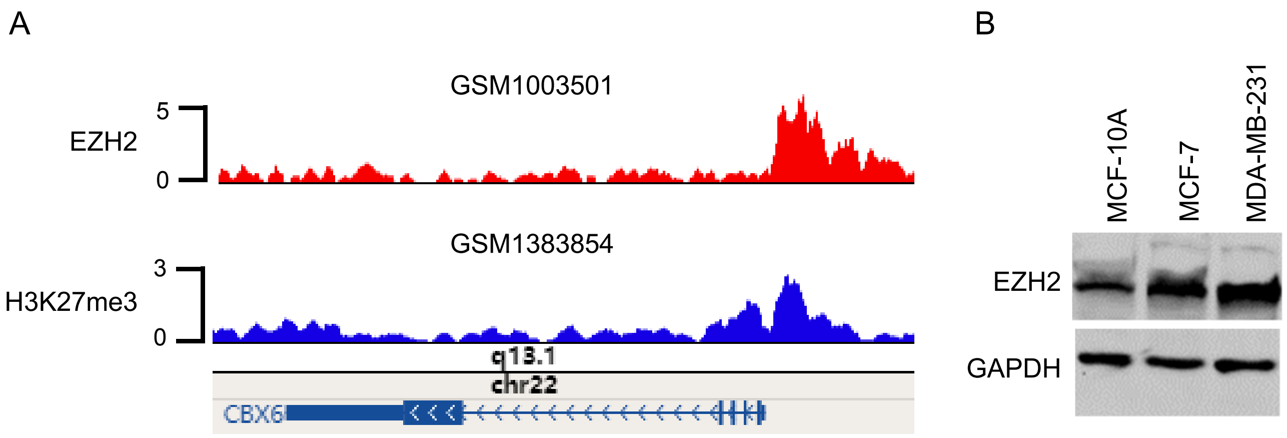

Figure S4

EZH2 negatively regulates CBX6 in breast cancer. (**A**) Binding patterns of EZH2 and H3K27me3 at the CBX6 locus in human mammary epithelial cells (HMEC). The peaks are visualized with the WashU Epigenome Browser. The Gene Expression Omnibus (GEO) accession number for the EZH2 ChIP-Seq dataset is GSM1003501, the GEO accession number for the H3K27me3 Chip-Seq dataset is GSM1383854. (**B**) EZH2 expression levels in breast cancer cell lines MCF-7, MDA-MB-231 and non-tumorigenic breast epithelial (MCF10A) cells were examined by western blot analysis. GAPDH was used as a loading control.

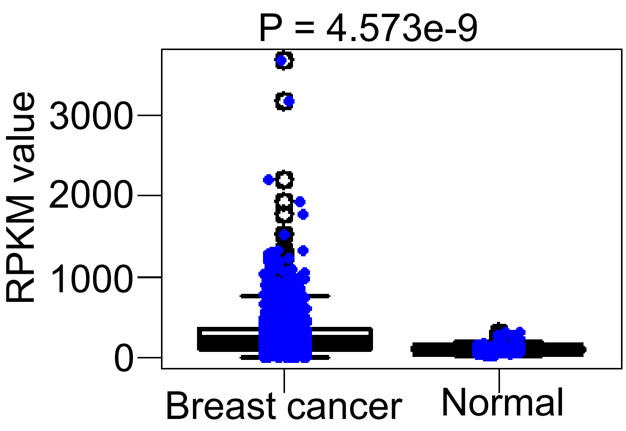

Figure S5

mRNA expression of Bone Marrow Stromal cell antigen 2 (*BST2*) in breast cancer versus normal breast tissues. The data are retrieved from the RNA-Seq datasets of The Cancer Genome Atlas (TCGA). The expression values are presented as Reads Per Kilobase of transcript per Million mapped reads (RPKM). A t-test was performed to determine significant differences in BST2 expression between normal breast tissue controls and breast cancer samples. P value is indicated at the top of the graph.

Supplementary Table S1. The primers used in the study.

CBX6 is negatively regulated by EZH2 and plays a potential tumor suppressor role in breast cancer

Houliang Deng1, Xiaowen Guan1, Longcai Gong1, Jianming Zeng1, Hongjie Zhang1, Mike Y. Chen2, Gang Li1*

**qRT-PCR Primers**	
hCBX6 F	aaacggcggatccgaaagggac
hCBX6 R	gctgcaatgagccgcgagtc
hEZH2 F	ttgccaagagagccatccag
hEZH2 R	cagctgtttcagaggagggg
hBST-2 F	atgtcacccatctcctgcaac
hBST-2 R	gcatccagggaagccattag
hGAPDH F	aggtgaaggtcggagtcaac
hGAPDH R	atctcgctcctggaagatgg
**ChIP Primers**	
CBX6 promoter F	ggcaacagcaactgcatttc
CBX6 promoter R	accctctccttgcctcttaatc
BST-2 promoter #1 F	caccacgcaactgtgcaacctc
BST-2 promoter #1 R	gccgtgccagcctgtattcatc
BST-2 promoter #2 F	tggacttgacctcgcctac
BST-2 promoter #2 R	cccattatcggcacccaac
BST-2 promoter #3 F	ctggcctccaatgtgcaattc
BST-2 promoter #3 R	aagacacgacctttgcctgac
**Cloning Primers**	
hEZH2 F	caccATGGGCCAGACTGGGAAGAAATC
hEZH2 R	TCAAGGGATTTCCATTTCTC

Supplementary_Full Length Gels and Blots
Deng H, et al. CBX6 is negatively regulated by EZH2 and plays a potential tumor suppressor role in breast cancer

Figure 1B

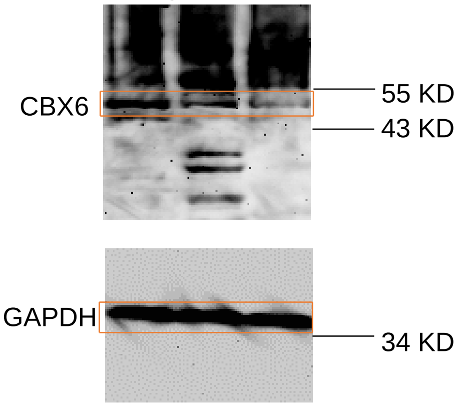

Figure 2A

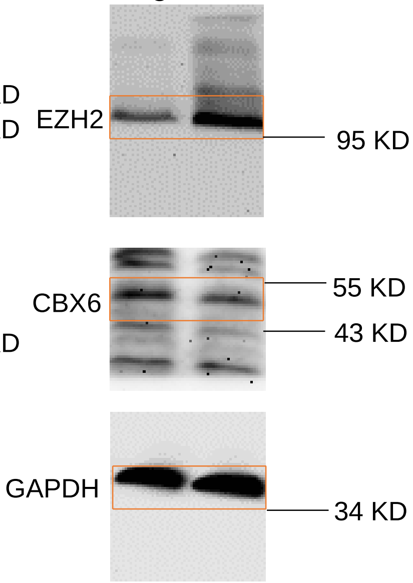

Figure 2B

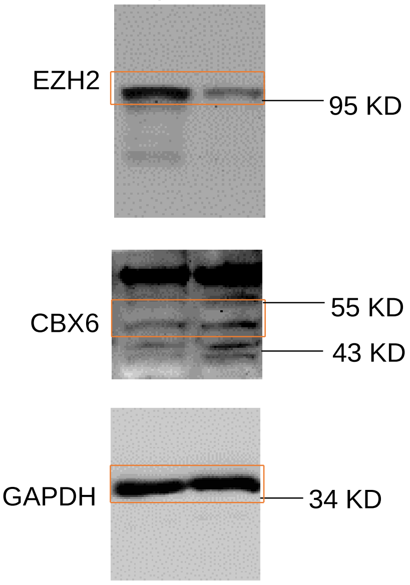

Figure 3A

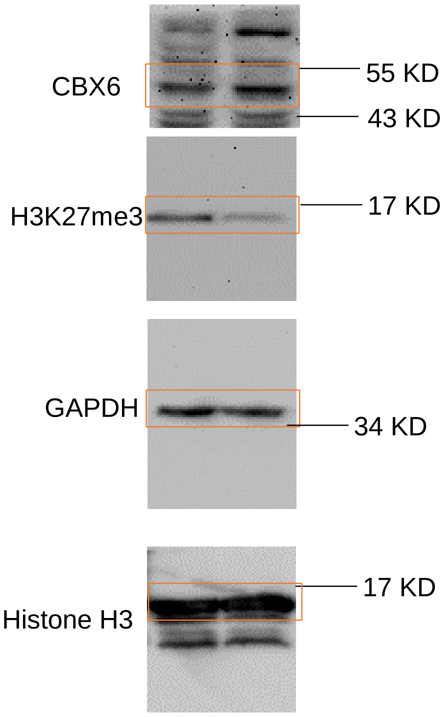

Figure 3B

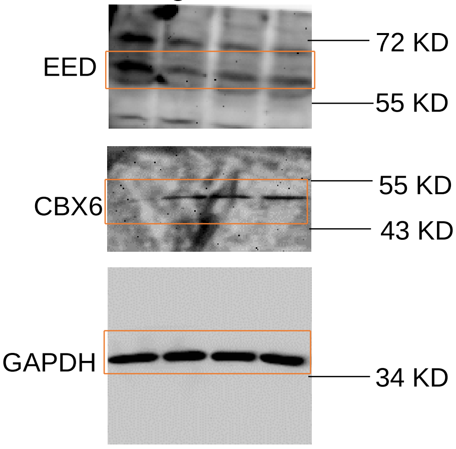

Figure 4A

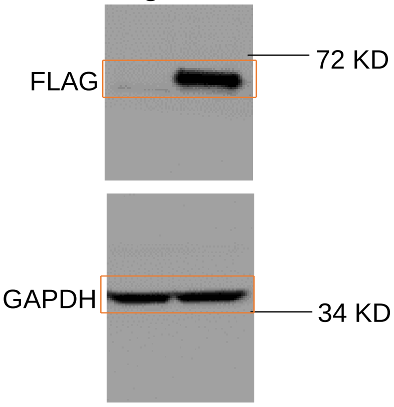

Figure 7A

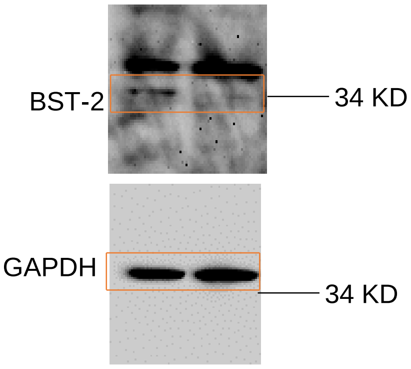

Supplement: Supplementary file 1 — Supplementary Information [file 41598_2018_36560_MOESM1_ESM.pdf]
